# A Comparative Study of Left Atrial Function Index of Hypertensive Heart Failure Patients Versus Controls in a Teaching Hospital, Sub-Saharan Africa

**DOI:** 10.7759/cureus.32954

**Published:** 2022-12-26

**Authors:** Anthony G Kweki, Ejiroghene M Umuerri, Fredrick I Aigbe, Henry O Aiwuyo, Austine O Obasohan

**Affiliations:** 1 Internal Medicine/Cardiology, Delta State University Teaching Hospital, Oghara, NGA; 2 Medicine, Delta State University, Abraka, NGA; 3 Internal Medicine, Brookdale University Hospital Medical Center, Brooklyn, USA; 4 Medicine, College of Medical Sciences, University of Benin, Benin City, NGA; 5 Internal Medicine/Cardiology, University of Benin Teaching Hospital, Benin City, NGA

**Keywords:** hfpef, hfref, hypertension, hypertensive hf, la function index

## Abstract

Background and aim: The left atrium contributes significantly to the left ventricular filling as it functions as a reservoir, conduit, and pump. These functions are referred to as the phasic function of the left atrium and they are assessed using left atrial volumes. The left atrial function index on the other hand is a rhythmic independent composite index which is a better marker of left atrial function. The study therefore aimed at comparing left atrial function (using the left atrial function index) among hypertensive heart failure patients, patients with hypertension but not in heart failure, and normotensive patients.

Method: The study was a cross-sectional analytical study that was carried out at Delta State University Teaching Hospital, Nigeria. A total of 80 hypertensive heart failure patients, 80 hypertensive, and 40 normotensive patients who met the inclusion criteria were recruited from the cardiology clinics using the convenience sampling method. The left atrial function index was determined using the volumetric method. Significance was assessed at *p *< 0.05.

Result: The left atrial function index (21.13 ± 8.83 versus 42.28 ± 10.40 versus 50.47 ± 14.37, *p *= 0.001) of the hypertensive heart failure group was significantly lowest when compared with the hypertensive (*p *< 0.001) and normotensive (*p *< 0.001) groups. Although the left atrial function index of the hypertensive group (42.28 ± 10.40) was lower than the normotensive group (50.47 ± 14.37), it was however not found to be significant (*p *= 0.12). Also, the left atrial function index was significantly (*p *= 0.001) worse among the patients with heart failure with reduced ejection fraction (13.5 ± 5.94) compared to heart failure with preserved ejection fraction (40.81 ± 12.12).

Conclusion: Left atrial function index was lowest among hypertensive heart failure patients compared with hypertensive and normotensive cohorts, and it was worse among heart failure with reduced ejection fraction patients. However, there was no significant difference between the left atrial function index of the hypertensive and normotensive groups. As a result, we recommend that the left atrial function index should be incorporated into the routine echocardiographic assessment of patients in our day-to-day clinical practice and large studies should be carried out to determine the cut-off value for the left atrial function.

## Introduction

In heart failure (HF), left atrium (LA) dysfunction is a mediator of impaired cardiac dynamics, and its structural and functional changes have different clinical implications [1,2]. The dilatation of the LA leads to mechanical failure which frequently culminates in atrial fibrillation. Moreover, LA mechanical failure is accompanied by LA endocrine failure and LA regulatory failure contributing to neurohumoral overactivity, vasoconstriction, and volume overload (global LA failure) [3]. The LA is central to the heart’s adaptive mechanism and it is an independent prognostic marker in patients with HF with reduced ejection fraction (EF); and a powerful predictor of adverse cardiovascular outcomes [4,5]. Most stressors of left atrial myocytes impose excess pressure and/or volume load on the LA, which responds to a range of adaptive and maladaptive processes. These responses include myocyte growth, hypertrophy, necrosis, and apoptosis; alterations in the composition of the extracellular matrix; recalibration of energy production and expenditure; changes in the expression of cellular ionic channels and atrial hormones; and reversal to a fetal gene program. The magnitude of LA alteration varies and is dependent on the type, severity, and duration of exposure to the varying stressors [6].

The LA serves as a reservoir, conduit, and pump and it exerts a profound effect on left ventricular (LV) filling and overall cardiovascular function [7]. These roles are referred to as the phasic function of the LA. Apart from providing a third of the cardiac output, it also acts as the barometer of the left ventricles, a marker of chronicity of cardiac diseases, and HF prognosis. Left atrial function index (LAFI), on the other hand, is a rhythmic-independent and composites index that better assesses the LA function and is inversely associated with age, hypertension, cardiovascular diseases, and positively associated with markers of cardiac remodeling [8]. It incorporates the fraction of blood that enters the left ventricle during early diastolic filling, the LV outflow tract velocity time integral, and the left atrial volume index (LAVI) to assess the LA function. This index has been demonstrated to represent the global function of the LA.

In Africa, some efforts have been made to assess LA function using phasic LA volumes. However, studies on LA function using LAFI are lacking even though it better represents a marker of pathological LA remodeling when compared to LA phasic volumes [8]. In addition, it is a significant early indicator of LV dysfunction even when EF is preserved [9]. As a result, this study aimed to compare LA function using LAFI among hypertensive heart failure (HHF) patients, patients with hypertension but not in HF, and normotensive patients.

## Materials and methods

The study was a comparative, cross-sectional, and analytical study carried out at Delta State University Teaching Hospital (DELSUTH), Oghara, Delta State, Nigeria, between June 2020 and June 2021.

The study population includes 80 patients who presented consecutively to the cardiology outpatient clinic for HHF (group 1), and this was age- and sex-matched with the hypertensive patient without HF (group 2), and normotensive patient (group 3). The inclusion criteria for the group 1 population were patients that are 18 years old and above who gave consent, irrespective of their left ventricular ejection fraction (LVEF), functional class, and whether they presented with acute or chronic HF. For group 2, inclusion criteria include those that are 18 years old and above who gave consent, irrespective of their blood pressure (BP) duration and control. The inclusion criteria for group 3 include healthy individuals aged 18 years or above who gave consent and are without any known medical or cardiovascular disease. For all the groups, those with suboptimal echocardiographic images (defined as non-visualization of at least two of six segments in the standard apical echocardiographic views), atrial fibrillation, atrial flutter, who refused or are unable to give consent, comorbidities (such as chronic kidney disease (eGFR<60mls/min/1.73m^2^), chronic obstructive pulmonary disease, valvular heart disease, diabetic mellitus, retroviral disease), or cancer and autoimmune disorders (such as systemic lupus erythematosus, scleroderma) were excluded.

HHF was defined as HF patients that have a clinical history of hypertension irrespective of their current BP status or persistent BP ≥140/90mmHg in those who are not previously known hypertensive and/or on antihypertensive medications [10,11]. Hypertension was defined as systolic BP ≥140mmHg and diastolic BP ≥90 mmHg or patients on antihypertensive medications [12].

Ethical approval was obtained from the Health Research and Ethics Committee of DELSUTH (Institutional Review Board number: HREC/PAN/2019/0313). Informed consent was obtained from participants and was duly signed/thumb-printed before they were recruited for the study. Confidentiality and the right to exit from the study at any time were maintained.

A semi-structured interviewer-administered questionnaire was used to obtain and record information on biodata, medical history, electrocardiography, and echocardiography. A transthoracic echocardiogram (Xario diagnostics ultrasound system model SSA-660A, Toshiba Medicals, probe frequency of 3.5MHz) with electrocardiography (ECG) gating was performed by the researcher at the time of recruiting patients.

LA function was assessed by the LAFI. To assess the LA function by the volumetric method, LA volumes were measured at different time points of the cardiac cycle: maximal LA volume (LAVmax) at the end of the T-wave on ECG, just before the opening of the mitral valve; minimal LA volume (LAVmin) at beginning of QRS-complex, just at the closure of the mitral valve; and preceding LA contraction volume (LAVpreA) at the beginning of P-wave [6]. The velocity time integral of the left ventricular outflow tract (LVOT-VTI) was determined by placing the sample volume of the pulse wave Doppler in the LVOT-VTI and the velocity time integral of spectral tracing was measured. The left atrial emptying fraction was determined by the difference between LA end-systolic volume (LAESV) and LA end-diastolic volume (LAEDV), all divided by the LAESV. LAFI was then calculated with the formula [9]:

LAFI = (LA emptying fraction x LVOT-VTI)/LAESV indexed to BSA

where;

LAFI = left atrial function index

LVOT-VTI = velocity time integral of the left ventricular outflow tract (cm),

LAESV = maximal left atrial volume in end-systole (cc)

BSA = body surface area (ml/m^2^)

LA emptying fraction = (LAESV-LAEDV)/LAESV

LA conduit function: the LA conduit function was assessed as follows:

LA passive emptying volume = LA maximum volume-LA pre ‘A’ wave volume;

LA passive emptying fraction = passive emptying volume/LA maximum volume x 100%;

Data was collated and analyzed with Statistical Product and Service Solutions (SPSS) (IBM SPSS Statistics for Windows, Version 22.0, Armonk, NY) [13]. The questionnaire used to obtain data from participants was checked for correctness. Continuous data such as height, weight, body mass index (BMI), and echocardiographic parameters (LAFI, LA emptying fraction, LA passive emptying fraction, and LA active emptying fraction, etc.) were presented as means and standard deviation, and the relationship among the variables was assessed with analysis of variance (ANOVA). Post-hoc analysis of LAFI among the three cohorts was assessed using Bonferroni. Significance was assessed at *p *< 0.05.

## Results

Baseline characteristics of study participants

Among the three groups, the hypertensive individuals had significantly higher weight (78.88 ± 11.46 versus 89.79 ± 14.07 versus 76.20 ± 13.39, p=0.001) and BMI (28.55 ± 48.2 versus 32.78 ± 4.74 versus 28.54 ± 4.84, p=0.001) but the waist circumference (96.25 ± 10.32 versus 99.31 ± 10.30 versus 99.97 ± 11.15, p=0.08) and waist-hip ratio (96.25 ± 10.32 versus 99.31 ± 10.30 versus 99.97 ± 11.15, p=0.640) were not statistically significantly different. The pulse rate (87.81 ± 19.56 versus 76.36 ± 12.91 versus 74.62 ± 9.52, p=0.001) was significantly higher among the HHF patients. However, the systolic blood pressure (125 ± 19.5 versus 147 ± 20.92 versus 116 ± 19.60, p=0.001), diastolic blood pressure (79 ± 11.52 versus 85 ± 12.06 versus 74 ± 6.03, p=0.001), pulse pressure (46.37 ± 15.43 versus 61.89 ± 19.40 versus 50 ± 19.70, p=0.001), and mean arterial pressure (94.98 ± 12.74 versus 106.3 ± 12.62 versus 88.08 ± 8.13, p=0.001) were significantly higher among the hypertensive group. Table 1 shows the baseline characteristics of the study participants.

**Table 1:** Baseline characteristics of the study participants (n=200) BSA - body surface area, BMI - body mass index, WC - waist circumference, WHR - waist-hip ratio, PR - pulse rate, SBP - systolic blood pressure, SBP - diastolic blood pressure, PP - pulse pressure, and MAP - mean arterial blood pressure. Kg/m2- kilogram per meter square; bpm - beats per minute; mmHg - millimeters of mercury, HHF - hypertensive heart failure, SD - standard deviation *Significant at p<0.05, **Compared using One-way Analysis of Variance (ANOVA)

Clinical parameters	Study Participants (Mean±SD)			F**	p-value
	Test Group 1 (HHF) (n=80)	Group 2 (Hypertensive) (n=80)	Group 3 (Normotensive) (n=40)		
Age (years)	62.15±7.30	61.95±7.24	62.13±7.37	0.192	0.83
Weight (kg)	78.88±11.46	89.79±14.07	76.20±13.39	16.71	0.001*
Height (m)	166.70±9.52	165.79±11.90	166.73±7.85	0.22	0.800
BSA (m^2^)	1.91±0.15	2.03±0.21	1.56±0.18	10.86	0.001*
BMI (kg/m^2^)	28.55±4.82	32.78±4.74	28.54±4.84	18.76	0.001*
WC (mm)	96.25±10.32	99.31±10.30	99.97±11.15	2.52	0.08
WHR	0.92 ± 0.05	0.92±0.05	0.92±0.05	0.46	0.64
PR (bpm)	87.81±19.56	76.36±12.91	74.62±9.52	20.19	0.001*
SBP (mmHg)	125±19.50	147±20.92	116±19.60	41.96	0.001*
DBP (mmHg)	79±11.52	85±12.06	74±6.03	17.22	0.001*
PP (mmHg)	46.37±15.43	61.89 ± 19.40	50±19.70	22.25	0.001*
MAP (mmHg)	94.98±12.74	106.3±12.62	88.08±8.13	35.75	0.001*

LAFI of study participants

LAFI (21.13 ± 8.83 versus 42.28 ± 10.40 versus 50.47 ± 14.37, p=0.001) was significantly lower among the HHF group compared to the other groups. Left atrial total emptying volume (LAtEV) (17.30 ± 7.70 versus 28.17 ± 10.22 versus 20.16 ± 7.60, p=0.001) and left atrial total emptying fraction (LatEF) (30.31 ± 12.36 versus 51.43 ± 14.36 versus 49.29 ± 10.07, p=0.001) were significantly lower among the HHF group. Although the left atrial passive emptying volume (LApEV) (10.03 ± 7.63 versus 13.25 ± 8.37 versus 10.68 ± 4.95, p=0.23) was not significantly lower among the three groups of patients, left atrial passive emptying fraction (LApEF) (16.54 ± 7.57 versus 23.77 ± 10.80 versus 26.46 ± 9.96, p=0.022) and LA conduit volume (LA CV) (41.63 ± 14.65 versus 55.09 ± 14.36 versus 64.61 ± 12.91, p=0.001) were significantly lower among the HHF. The left atrial active emptying volume (LAaEV) (7.27 ± 2.59 versus 9.92 ± 7.14 versus 11.54 ± 9.07, p=0.025) and left atrial active emptying fraction (LAaEF) (14.36 ± 9.0 versus 26.39 ± 16.82 versus 33.66 ± 15.18, p=0.001) were significantly lower among the HHF compared to the other groups. Table 2 shows the LAFI of HHF patients compared to hypertensive and normotensive patients.

**Table 2:** Left atrial indices of study participants (n=200) LAVmax - maximal left atrial volume, LAVpreA - preceding left atrial contraction volume, LAVmin - minimal left atrial volume, LAtEV - left atrial total emptying volume, LAtEF - left atrial total emptying fraction, LApEV - left atrial passive emptying volume, LApEF - left atrial passive emptying fraction, LA CV - left atrial conduit volume, LAaEV - left atrial active emptying volume, LAaEF - left atrial active emptying fraction, LAFI - left atrial function index Kg/m2 - kilogram per meter square; mmHg - millimeters of mercury, ml/m2 - milliliters per meter square, SD - standard deviation, LAESVI - left atrial end-systolic volume index *Significant at p<0.05; **Compared using One-Way Analysis of Variance (ANOVA)

LA function indices	Study Participants (Mean±SD)			F**	p-value
	Test Group 1 (HHF) (n=80)	Group 2 (Hypertensive) (n=80)	Group 3 (Normotensive) (n=40)		
LAVmax (ml)	60.64±18.62	55.74±15.27	40.37±14.47	20.32	0.001*
LAVpreA (ml)	50.61±16.64	42.49±14.22	29.69±13.04	12.73	0.001*
LAVmin (ml)	43.34±16.76	27.57±12.10	20.16±7.62	47.68	0.001*
LAESVI (ml/m^2^)	32.55±10.43	28.23±8.43	21.27±7.80	20.51	0.001*
LAtEV (ml)	17.30±7.70	28.17±10.22	20.16±7.60	19.61	0.001*
LAtEF (%)	30.31±12.36	51.43±14.36	49.29±10.07	61.30	0.001*
LA CV (ml)	41.63±14.65	55.09±14.36	64.61±12.91	38.91	0.001*
LApEV (ml)	10.03±7.63	13.25±8.37	10.68±4.95	1.50	0.23
LApEF (%)	16.54±7.57	23.77±10.80	26.46±9.96	12.91	0.02*
LAaEV (ml)	7.27±2.59	13.92±7.14	11.54±9.07	41.67	0.03*
LAaEF (%)	14.36±9.0	36.39±16.82	33.66±15.18	75.29	0.001*
LAFI	21.13±8.83	42.28±10.40	50.47±14.37	34.55	0.001*

Post hoc analysis of LAFI among study participants

**Figure 1:**
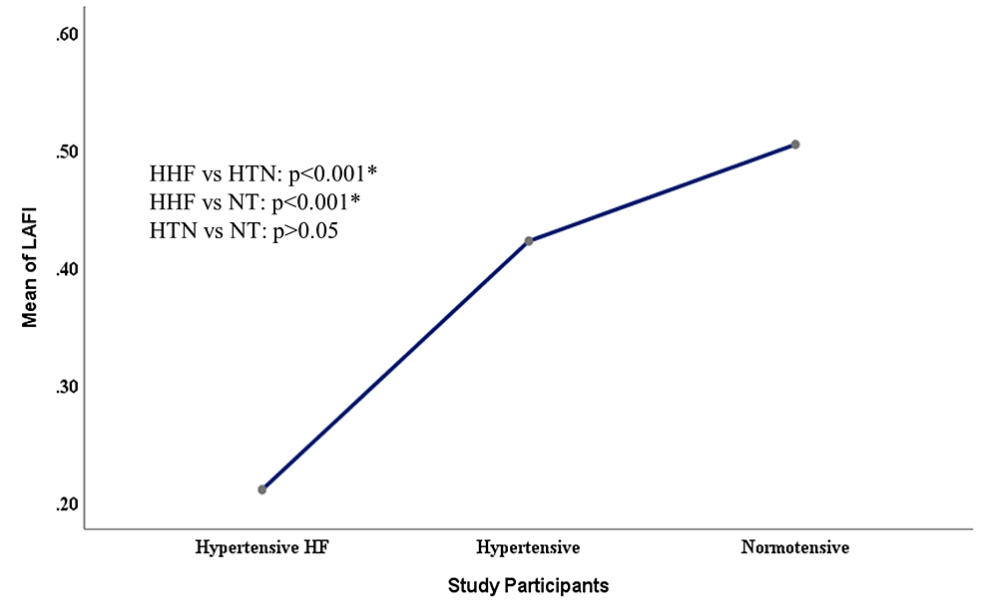
Mean plot showing the comparison of LAFI among study participants HHF - hypertensive heart failure, HTN - hypertensive, NT - normotensive, HF - heart failure, LAFI - left atrial function index *significant at p<0.05

Figure 1 showed a post hoc analysis of LAFI among the study participants using Bonferroni. It showed a significantly lower LAFI among the HHF group compared to hypertensive and normotensive groups (p=0.001) but there was no significant difference between the LAFI of hypertensive and normotensive groups (p=0.12).

LAFI of HHF based on LVEF

The LAFI was significantly lower among the HF with reduced ejection fraction (HFrEF) group compared to the HFpEF (13.32 ± 5.94 versus 40.81 ± 12.12, p=0.001). Among HHF patients, the LA reservoir function of HFrEF was significantly lower compared to HFpEF (LAtEV: 17.35 ± 8.12 versus 23.41 ± 10.48, p=0.001; LAtEF: 26.87 ± 11.25 versus 33.57 ± 12.62, p=0.01). The LA conduit function was found to be lower among the HFrEF compared to HFpEF (LApEV: 14.51 ± 7.94 versus 15.18 ± 8.77, p=0.67; LApEF: 22.32 ± 10.87 versus 29.74 ± 13.62, p=0.09) but these findings were not significant. The LAaEV (2.84 ± 3.33 versus 8.24 ± 7.80, p=0.001) and LAaEF (5.81 ± 2.10 vs 23.01 ± 17.76, p=0.001) were significantly lower among the HFrEF compared to HFpEF. Table 3 shows the LAFI of HHF based on EF.

**Table 3:** Left atrial indices of hypertensive heart failure based on LVEF (n=80) LVEF - left ventricular ejection fraction, LAVmax - left atrial maximum volume, LAVpre’A’ - left atrial pre atrial contraction volume, LAVmin - left atrial minimum volume, LAESVI - left atrial end-systolic volume index, LAtEV - left atrial total emptying volume, LAtEF - left atrial total emptying fraction, LApEV - left atrial passive emptying volume, LApEF - left atrial passive emptying fraction, LA CV - left atrial conduit volume, LAaEV - left atrial active emptying volume, LAaEF - left atrial active emptying fraction, LAFI - left atrial function index *Significant at p<0.05; **Compared using independent sample t-test Reduced LVEF: Reduced Left Ventricular Ejection Fraction (<50%) Preserved LVEF: Preserved Left Ventricular Ejection Fraction (≥50%)

LA function indices	Hypertensive heart failure (Mean±SD)		t test**	p-value
	Reduced LVEF (n=39)	Preserved LVEF (n=41)		
LAVmax (ml)	66.13±14.54	55.41±20.63	2.67	0.01*
LAVpreA (ml)	51.62±13.74	39.90±17.29	3.37	0.001*
LAVmin (ml)	48.78±13.94	38.17±17.72	2.97	0.01*
LAESVI (ml/m^2^)	35.88±8.60	29.40±11.13	2.91	0.01*
LAtEV (ml)	17.35±8.12	23.41±10.48	3.37	0.001*
LAtEF (%)	26.87±11.25	33.57±12.62	2.50	0.01*
LA CV (ml)	36.36±12.40	46.63±15.00	3.33	0.001*
LApEV (ml)	14.51±7.94	15.18±8.77	0.43	0.67
LApEF (%)	22.32±10.87	29.74±13.62	3.17	0.09
LAaEV (ml)	2.84±3.33	8.24±7.80	4.22	0.001*
LAaEF (%)	5.81±2.10	23.01±17.76	5.95	0.001*
LAFI	13.32±5.94	40.81±12.12	7.28	0.001*

## Discussion

In our study, the LAFI of HHF and hypertensive patients differ significantly, being worse in HHF patients. The hypertensive group had lower LAFI compared to the normotensive group, but this finding was not of statistical significance. The reduced level of LAFI among HHF may be attributed to progressive LA dilation, poor LA contractile function, and impaired atrial compliance which occurs as hypertensive patients progressively develop hypertensive heart disease. In other words, HF symptoms develop among hypertensive individuals when the LA losses its compensatory mechanism and becomes dysfunctional leading to pulmonary congestion. The Framingham offspring study [8] reported a mean LAFI of 34.5 ± 12.7 but our study reported an average value of 21.13 ± 18.83, 42.28 ± 12.40, 50.47 ± 14.37 among the HHF group, hypertensive without HF and normotensive groups respectively. They [8] also noted that LAFI was significantly higher (37.5 ± 11.6) in a subgroup of participants free of cardiovascular disease and cardiovascular disease risk factors compared with those with either of these conditions (34.5 ± 12.2). Our study also demonstrated similar findings with significantly lower LAFI (21.13 ± 8.83 versus 42.28 ± 10.40 versus 50.47 ± 14.37, p=0.001) among the HHF group compared to the controls. This may indicate that as patients developed hypertensive heart disease, the LA function becomes dysfunctional as a result of impaired atrial compliance and contractile function. Similarly, another study [14] reported that LA active emptying was higher in a patient with mild diastolic dysfunction compared with patients with normal diastolic function and it gradually decreased as diastolic dysfunction worsen in severity. In that study [14], active LA emptying fraction was also found to be lower among HHF patients compared with hypertensive patients, with the former having a higher ratio of the early trans-mitral inflow to the early trans-annular velocity (E/E') suggesting worsening LV diastolic dysfunction. The authors [14] used active LA emptying fraction to assess the LA function instead of LAFI which is a better index of the LA function because it incorporates LA emptying fractions and other components of LA mechanics.

A Chinese study [15] that evaluated LA function with the LAFI in patients with essential hypertension reported that LAFI in the hypertensive group was significantly reduced compared with normal controls (0.53 ± 0.19 versus 0.79 ± 0.23, p<0.05). Similarly, our study also demonstrated lower LAFI among the hypertensive cohort compared to the control (42.28 ± 10.40 versus 50.47 ± 14.37) but this was not found to be significant (p=0.12). The non-significant of LAFI in our study may be due to the fact we were unable to establish patients who are treatment-naïve versus those who are not treatment-naïve. However, there is a trend that hypertensive individuals tend to have lower LAFI as depicted in our study and corroborated by other authors [15].

Another study [16] stated that HFrEF and HF with preserved ejection fraction (HFpEF) patients were associated with LA dysfunction and worse among patients with HFrEF. We also found significantly lower LAFI among HFrEF (13.32 ± 5.94) compared to HFpEF (40.81 ± 12.12). Global systolic dysfunction including LA dysfunction which occurs more in HFrEF may account for these findings. This is because LA emptying fraction, contraction, and size will be more affected. It was also noted by another study [17] that the LA function of HFpEF compared to hypertensive and normotensive patients was significantly decreased (p=0.005). This further buttress the fact that as the left ventricular compliance worsen, there is a decrease in the LA function which has been demonstrated in our study. Similar findings were reported that LA emptying fractions were significantly lower among HFrEF, HFpEF, and diastolic dysfunction patients versus normal controls [18].

Limitation of study

Transthoracic echocardiography was used for the assessment of left atrial function instead of transoesophageal echocardiography which better assesses left atrial function.

There were limited funds for carrying out specific tests to rule cofounders and as such results were retrospectively obtained from patients' case notes.

## Conclusions

The LAFI is inversely related to hypertensive heart disease categories making it an excellent tool for assessing individuals with HHF. We recommended that LAFI should be incorporated into the routine assessment of patients in our day-to-day clinical practice. We also propose that large prospective studies on LAFI should be carried out to determine partition value for the assessment of left atrial function and HF prognostication.
